# Payload Release Profile and Anti-Cancer Stem Cell Properties of Compositionally Different Polymeric Nanoparticles Containing a Copper(II) Complex

**DOI:** 10.3390/molecules28062506

**Published:** 2023-03-09

**Authors:** Ginevra Passeri, Joshua Northcote-Smith, Kogularamanan Suntharalingam

**Affiliations:** School of Chemistry, University of Leicester, Leicester LE1 7RH, UK

**Keywords:** polymeric nanoparticles, cancer stem cells, poly lactic-*co*-glycolic acid, polyethylene glycol, copper

## Abstract

Cancer stem cells (CSCs) are linked to tumour relapse and metastasis, the main reason for cancer-related deaths. The application of polymeric nanoparticles as drug delivery systems to target CSCs is relatively unexplored. Here, we report the encapsulation of a CSC-potent copper(II) complex **1** by two compositionally different methoxy poly(ethylene glycol)-*b*-poly(D,L-lactic-co-glycolic) acid (PEG–PLGA) copolymers. Specifically, we used PEG–PLGA (5000:10,000 Da, 1:1 LA:GA) and PEG–PLGA (5000:10,000 Da, 4:1 LA:GA) polymers to prepare spherical nanoparticle formulations **1:1 NP^15^** and **4:1 NP^15^**, respectively, both with a 15% feed of **1**. The two formulations show distinct biophysical and in vitro properties. For example, (i) **4:1 NP^15^** displays a slower payload release profile than **1:1 NP^15^** in physiologically relevant solutions, (ii) **4:1 NP^15^** exhibits statistically greater potency towards breast CSCs than bulk breast cancer cells grown in monolayers, whereas **1:1 NP^15^** is equally potent towards breast CSCs and bulk breast cancer cells, and (iii) **4:1 NP^15^** shows significantly greater potency towards three-dimensionally cultured mammospheres than **1:1 NP^15^**. This study shows that the release profile and anti-breast CSC properties of PEG–PLGA nanoparticle formulations (containing **1**) can be perturbed (and possibly controlled) by modifying the proportion of glycolic acid within the PLGA component.

## 1. Introduction

Cancer stem cells (CSCs) are a small subset of tumour cells implicated in cancer progression, metastasis, therapy resistance, and reoccurrence [1,2]. Removing CSCs at safe doses could lead to durable patient outcomes and life-long remission [3]. There are several ongoing research programmes aimed at developing safe anti-CSC agents (comprising small molecules, biologics or nano-sized systems) that target established CSC characteristics such as overexpressed cell surface markers, dysregulated signalling pathways, and overactive organelles [4,5]. Despite these efforts, there is currently no clinically approved drug formulation that can remove CSCs (of any tissue type) when administered at a concentration that does not induce significant systemic toxicity [6]. In terms of small-molecule anti-CSC drug development, the overwhelming majority of academic and industrial endeavours have focused on purely organic molecules [4,5]. We and others have shown, over the last 8 years, that metal-containing compounds can also display clinically relevant CSC potencies [7,8,9].

**Figure 1:**
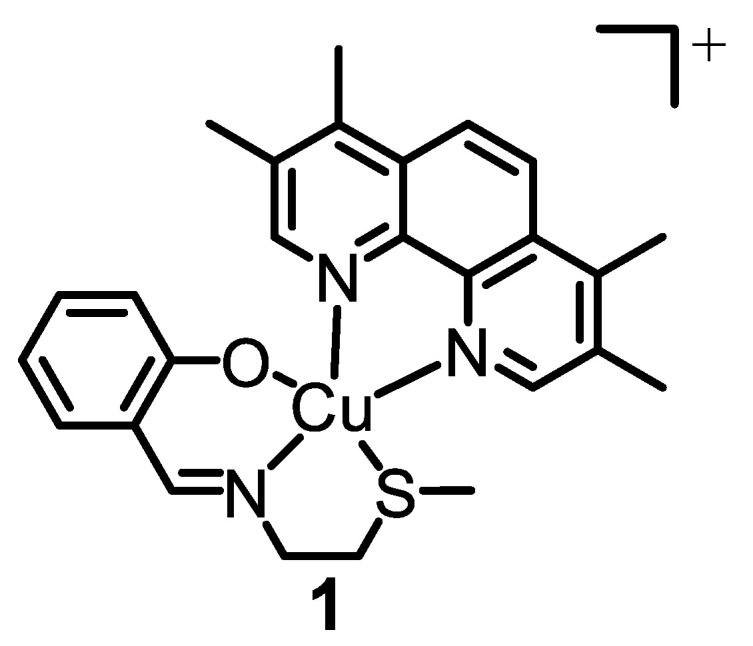
Chemical structure of a copper(II) complex **1** capable of killing breast CSCs via cytotoxic and immunogenic pathways. The copper(II) complex **1** will serve as the payload for the nanoparticle formulations prepared in this study.

The intracellular redox state in certain CSCs is meticulously regulated [10,11]. We have previously used reactive oxygen species (ROS)-generating endogenous metal (copper and manganese) coordination compounds to perturb this equilibrium and effect breast CSC-selective toxicity [12,13,14,15]. The most effective metal complex developed by our research group thus far is a copper(II) complex **1** bearing a 4,7-diphenyl-1,10-phenanthroline ligand and an *O*,*N*,*S*-donating Schiff base ligand (see Figure 1 for chemical structure) [16]. Remarkably, the copper(II) complex **1** is able to kill breast CSCs grown in monolayer and three-dimensional cell cultures via cytotoxic and immunogenic mechanisms [16]. Specifically, **1** generates ROS in the ER of breast CSCs, and thus induces ER stress, apoptosis, and immunogenic cell death (ICD) [16]. Despite the very promising anti-CSC properties of **1** in vitro, further translation was not possible as **1** is susceptible to reduction, under biological conditions, which leads to undesirable structural transformations. To overcome this challenge, we employed methoxy poly(ethylene glycol)-*b*-poly(D,L-lactic-co-glycolic) acid (PEG–PLGA) (5000:20,000 Da, 1:1 LA:GA), a biodegradable amphiphilic copolymer, to encapsulate and deliver **1** in its intact form into breast CSCs [17]. The spherical PEG–PLGA nanoparticle formulation enhanced breast CSC uptake and improved potency towards bulk breast cancer cells and breast CSCs (grown in monolayer and three-dimensional cultures) relative to payload **1** [17]. Importantly, the PEG–PLGA nanoparticle formulation was able to evoke the same mechanism of action as payload **1**, including intracellular ROS elevation, ER stress, and ICD [17].

Here, we have sought to determine the anti-breast CSC properties of two new nanoparticle formulations comprising **1** and compositionally different PEG–PLGA copolymers. Specifically, PEG–PLGA (5000:10,000 Da, 1:1 LA:GA) and PEG–PLGA (5000:10,000 Da, 4:1 LA:GA) were used to encapsulate and deliver **1** into breast CSCs. The PLGA composition (lactic acid to glycolic acid ratio) is a determining factor in the rate of hydrolysis of the polymer and hence the rate of payload release from PEG–PLGA nanoparticles [18]. PLGA-containing nanoparticles with a higher proportion of glycolic acid undergo faster hydrolysis and payload release owing to their higher hydrophilicity [18,19,20]. Therefore, by reducing the proportion of glycolic acid within the PLGA component, payload release from PEG-PLGA nanoparticles can be attenuated and thus better controlled. In this study, we compare the payload release profile of two compositionally different PEG–PLGA nanoparticles (one with a 1:1 lactic acid to glycolic acid ratio and the other with a 4:1 ratio) within the context of anti-CSC drug delivery. As far as we are aware, this is the first study to attempt to control the release of an anti-breast CSC metal complex from PEG–PLGA nanoparticles.

## 2. Results and Discussion

**Figure 2:**
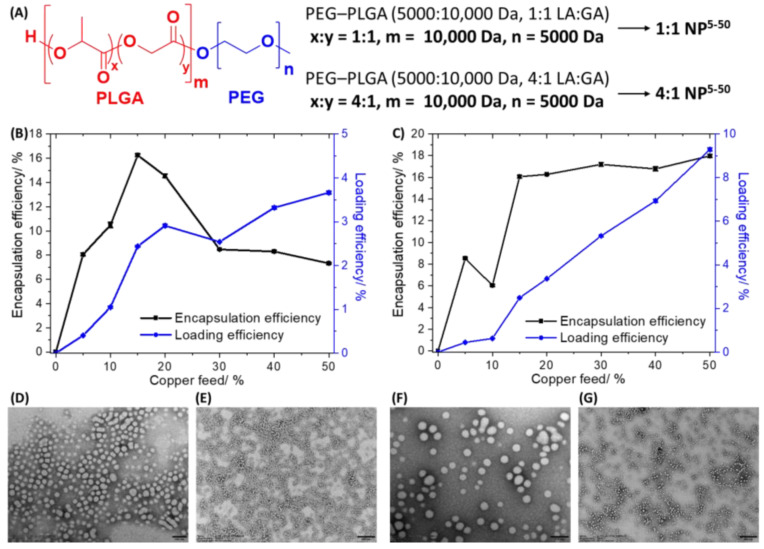
(**A**) Chemical structures of PEG–PLGA (5000:10,000 Da, 1:1 LA:GA) and PEG–PLGA (5000:10,000 Da, 4:1 LA:GA) polymers and the nomenclature used to describe the PEG–PLGA nanoparticles formulations prepared with the various feeds of **1**. The effect of feed variation on encapsulation and loading efficiency of **1** incorporated into (**B**) PEG–PLGA (5000:10,000 Da, 1:1 LA:GA) or (**C**) PEG–PLGA (5000:10,000 Da, 4:1 LA:GA) nanoparticles. TEM images of **1:1 NP^15^** suspended in water at (**D**) ×40,000 magnification, scale bar = 100 nm and (**E**) ×10,000 magnification, scale bar = 500 nm. TEM images of **4:1 NP^15^** suspended in water at (**F**) ×50,000 magnification, scale bar = 100 nm and (**G**) ×10,000 magnification, scale bar = 500 nm.

The copper(II) complex **1** was encapsulated into biodegradable and biocompatible PEG–PLGA (5000:10,000 Da, 1:1 LA:GA) and PEG–PLGA (5000:10,000 Da, 4:1 LA:GA) nanoparticles using the nanoprecipitation method. Figure 2A displays the chemical structures of the PEG–PLGA (5000:10,000 Da, 1:1 LA:GA) and PEG–PLGA (5000:10,000 Da, 4:1 LA:GA) polymers used to prepare the nanoparticles. The nanoprecipitation method involves dissolving **1** (0.5–5 mg) and the appropriate PEG–PLGA polymer (10 mg) in DMF and steadily adding this mixture to a vigorously stirring vessel of water. As the PEG–PLGA polymers used are amphiphilic they tend to self-assemble to form spherical nanoparticles with a hydrophilic exterior made up of PEG and a hydrophobic interior comprising PLGA. As **1** is a relatively hydrophobic compound (LogP = 2.01 ± 0.16) it will naturally become encapsulated into the hydrophobic core of the PEG–PLGA (5000:10,000 Da, 1:1 LA:GA) and PEG–PLGA (5000:10,000 Da, 4:1 LA:GA) nanoparticles. Different nanoparticle formulations of **1** and PEG–PLGA (5000:10,000 Da, 1:1 LA:GA) (**1:1 NP^5−50^**) or PEG–PLGA (5000:10,000 Da, 4:1 LA:GA) (**4:1 NP^5−50^**) were prepared by altering the feed (percentage of **1** to PEG–PLGA polymer in terms of mass) between 5% and 50%. Figure 2A depicts the nomenclature used to describe the PEG–PLGA nanoparticle formulations prepared with the various feeds of **1**. The amount of copper in each formulation was determined by inductively coupled plasma mass spectrometry (ICP-MS) after digestion by concentrated nitric acid, and this was used to calculate the encapsulation and loading efficiency of **1** and determine the most appropriate formulation for cell-based studies. The encapsulation efficiency is the amount of copper present in the final nanoparticle formulation relative to the amount of **1** used ×100, whereas loading efficiency is the amount of copper present in the final nanoparticle formulation relative to the amount of polymer used ×100. The change in encapsulation and loading efficiency of **1** with respect to feed for the PEG–PLGA (5000:10,000 Da, 1:1 LA:GA) and PEG–PLGA (5000:10,000 Da, 4:1 LA:GA) nanoparticles is presented in Figure 2B,C. The hydrophilic composition of the PEG–PLGA (5000:10,000 Da, 1:1 LA:GA) and PEG–PLGA (5000:10,000 Da, 4:1 LA:GA) polymers is different and hence the encapsulation and loading efficiency varies across the different feeds used. For the PEG–PLGA (5000:10,000 Da, 1:1 LA:GA) nanoparticles, the optimal encapsulation conditions were achieved at 15% feed (**1:1 NP^15^**) where the encapsulation efficiency was 16.25 ± 0.11% and the loading efficiency was 2.44 ± 0.02%. For the PEG–PLGA (5000:10,000 Da, 4:1 LA:GA) nanoparticles, according to the feed versus encapsulation efficiency plot, the encapsulation efficiency decreased from 5% to 10% feed, and then increased dramatically at 15% feed before remaining largely unchanged until 50% feed. According to the feed versus loading efficiency plot for the PEG–PLGA (5000:10,000 Da, 4:1 LA:GA) nanoparticles, the loading efficiency steadily increased with the increasing feed. Based on the encapsulation and loading efficiency data and taking into consideration the mass of **1** that would be needed for preparing each batch of nanoparticles, 15% feed (**4:1 NP^15^**) was chosen as the most practical and efficient option to prepare PEG–PLGA (5000:10,000 Da, 4:1 LA:GA) nanoparticles containing sufficient **1** to induce a cytotoxic effect. At 15% feed (**4:1 NP^15^**) the encapsulation efficiency was 16.06 ± 0.14% and the loading efficiency was 2.49 ± 0.02%.

The optimal nanoparticle formulations **1:1 NP^15^** and **4:1 NP^15^** were characterised by dynamic light scattering (DLS) to have an average nanoparticle diameter of 78.63 ± 0.79 nm and 80.79 ± 3.56 nm, respectively (Figures S1 and S2). The polydispersity of **1:1 NP^15^** and **4:1 NP^15^** was 0.227 ± 0.005 and 0.123 ± 0.003, respectively (Figures S1 and S2). The average diameters of **1:1 NP^15^** and **4:1 NP^15^** were 23–39% higher than the corresponding empty PEG–PLGA (5000:10,000 Da, 1:1 LA:GA) (56.72 ± 0.47 nm, Figure S3) and PEG–PLGA (5000:10,000 Da, 4:1 LA:GA) (65.81 ± 0.24 nm, Figure S4) nanoparticles, implicative of successful encapsulation of **1** into the PEG–PLGA nanoparticles. The surface morphology and size distribution of **1:1 NP^15^** and **4:1 NP^15^** were assessed by transmission electron microscopy (TEM). The TEM images confirmed that **1:1 NP^15^** and **4:1 NP^15^** form relatively uniform spherical structures with an average size of 55.6 ± 6.4 nm and 60.2 ± 3.1 nm, respectively (Figure 2D–G). The average nanoparticle size determined for **1:1 NP^15^** and **4:1 NP^15^** using TEM analysis is in reasonable agreement with the DLS measurements.

Prior to conducting cell-based studies and establishing the release profile of the nanoparticle formulations, their solution stability in biologically relevant conditions was monitored over the course of 72 h. Once each nanoparticle formulation is prepared and characterised, it is stored in water (at 4 °C for a maximum of 3 days). For cell-based studies, the nanoparticle formulation is diluted to the working concentration(s) in cell culture media. Therefore, the solution stability of **1:1 NP^15^** and **4:1 NP^15^** was probed in water and Mammary Epithelial Cell Growth Medium (MEGM) over the course of 72 h at 37 °C (Figures S5 and S6). The average diameter of **1:1 NP^15^** and **4:1 NP^15^** was relatively constant in water (pH 7.4) over the course of 72 h, suggestive of good stability (Figures S5 and S6). The average diameter of **1:1 NP^15^** and **4:1 NP^15^** was relatively unaltered in MEGM (pH 7.4) for 24 h, suggestive of good stability (Figures S5 and S6). From 24 h to 72 h incubation in MEGM, a 2-fold and 1.9-fold increase in diameter was observed for **1:1 NP^15^** and **4:1 NP^15^**, respectively, indicative of aggregation or corona formation whereby biomolecules within MEGM bind to the surface of the nanoparticles (Figures S5 and S6). Overall, the solution stability studies indicate that **1:1 NP^15^** and **4:1 NP^15^** are stable in water for up to 72 h and in MEGM for 24 h. Given the relatively fast internalisation of 50–200 nm sized polymeric nanoparticles by dividing cells (in the order of a few hours) [21], the solution stability data for **1:1 NP^15^** and **4:1 NP^15^** bodes well for the potential delivery of **1** into breast CSCs in its intact form.

**Figure 3:**
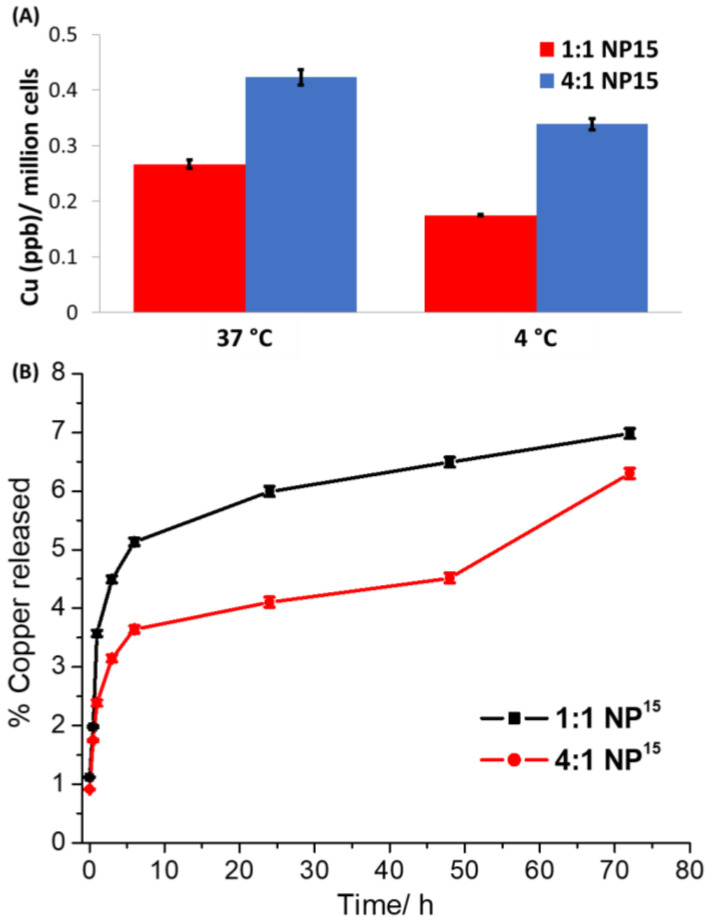
(**A**) Copper content in HMLER-shEcad cells treated with **1:1 NP^15^** and **4:1 NP^15^** (both 0.15 µM for 4 h) at 37 °C or 4 °C. (**B**) The amount of copper released from **1:1 NP^15^** and **4:1 NP^15^** upon incubation in PBS (pH 7.4) over the course of 72 h at 37 °C.

The ability of the nanoparticle formulations **1:1 NP^15^** and **4:1 NP^15^** to be internalised by breast CSCs (HMLER-shEcad cells) was investigated. HMLER-shEcad cells treated with **1:1 NP^15^** and **4:1 NP^15^** (both 0.15 µM for 8 h at 37 °C) were extracted, digested using nitric acid, and analysed for their copper content using ICP-MS. This method gives a good indication of the cellular uptake of **1:1 NP^15^** and **4:1 NP^15^** as both nanoparticle formulations contain **1** as the payload, which is a copper-containing compound. The nanoparticle formulations **1:1 NP^15^** (287.4 ± 9.0 ng of Cu/million cells) and **4:1 NP^15^** (279.5 ± 13.3 ng of Cu/million cells) were taken up to a similar extent by breast CSCs. This shows that the compositional difference (lactic acid to glycolic acid ratio) between **1:1 NP^15^** and **4:1 NP^15^** does not affect the amount of payload internalised by breast CSCs. For context, it is important to note that the endogenous amount of copper in HMLER-shEcad cells (untreated control cells) was 0.91 ± 0.04 ng of Cu/million cells. To decipher the mode of uptake of **1:1 NP^15^** and **4:1 NP^15^** by breast CSCs, studies were carried out at varying temperatures and in the presence of a specific uptake pathway inhibitor. HMLER-shEcad cells were treated with **1:1 NP^15^** and **4:1 NP^15^** (both 0.15 µM for 4 h) at 4 °C and 37 °C, and the copper content in the respective cells was measured by ICP-MS (Figure 3A). HMLER-shEcad cells treated with **1:1 NP^15^** and **4:1 NP^15^** at 37 °C contained 34% and 20% more copper, respectively, than the same cells treated with **1:1 NP^15^** and **4:1 NP^15^** at 4 °C. The temperature-dependent uptake observed for **1:1 NP^15^** and **4:1 NP^15^** suggests that both nanoparticle formulations are taken up by breast CSCs via an active process. Polymeric nanoparticles made up of PEG–PLGA are often internalised into cells by endocytosis [22]. To determine if **1:1 NP^15^** and **4:1 NP^15^** are taken up by breast CSCs via endocytosis, uptake experiments were carried out in the presence of a well-established endocytosis inhibitor, chloroquine (Figure S7). Specifically, HMLER-shEcad cells were pre-treated with chloroquine (100 μM for 2 h) and then treated with **1:1 NP^15^** or **4:1 NP^15^** (both 0.15 µM for 8 h at 37 °C), after which the cells were extracted, digested using nitric acid, and analysed for copper by ICP-MS. As expected, a significant decrease (*p* < 0.05) in **1:1 NP^15^** and **4:1 NP^15^** uptake was observed in the presence of chloroquine, indicating that **1:1 NP^15^** or **4:1 NP^15^** both enter breast CSCs cells via an endocytic mechanism. Overall, the cell uptake studies indicate that the compositional difference (lactic acid to glycolic acid ratio) between **1:1 NP^15^** and **4:1 NP^15^** does not significantly influence the amount or mode of cell uptake by breast CSCs.

The aptitude of polymeric nanoparticles to release their payload once internalised by cells is a determining factor in terms of efficacy. The ability of **1:1 NP^15^** or **4:1 NP^15^** to release its payload **1** in physiologically relevant conditions (PBS, pH 7.4 at 37 °C) over the course of 72 h was determined by time course dialysis studies (Figure 3B). The release profile of **1:1 NP^15^** or **4:1 NP^15^** both display an initial burst release of their payload in the first 1 h of incubation. From 1 h to 72 h incubation, there is a clear difference between the release profile of **1:1 NP^15^** and **4:1 NP^15^**. The **1:1 NP^15^** formulation releases its payload at a faster rate and to a greater amount than the **4:1 NP^15^** formulation. This is expected as the **1:1 NP^15^** formulation contains a higher proportion of glycolic acid than the **4:1 NP^15^** formulation and hence is likely to undergo faster hydrolysis and payload release. In sodium acetate buffer (pH 5.2 at 37 °C), **1:1 NP^15^** and **4:1 NP^15^** released **1** to a significantly greater extent than in PBS (Figure S8). A difference in the release profile of **1:1 NP^15^** and **4:1 NP^15^** over the course of 72 h was observed in sodium acetate buffer; however, the difference was less stark than in PBS. A slightly faster rate of release was observed for the **1:1 NP^15^** formulation over the **4:1 NP^15^** formulation. Taken together, this shows that the release profile of PLGA-PEG nanoparticles containing the CSC-active copper(II) complex **1** can be altered (and possibly controlled) by manipulating the proportion of glycolic acid within the PLGA component. The dialysis studies also show that both **1:1 NP^15^** and **4:1 NP^15^** are able to release **1** in physiologically relevant and mildly acidic solutions and thus implies that **1:1 NP^15^** and **4:1 NP^15^** are capable of releasing **1** upon endocytic internalisation by breast CSCs.

The potency of the nanoparticle formulations **1:1 NP^15^** and **4:1 NP^15^** towards breast CSC-enriched (HMLER-shEcad) and breast CSC-depleted (HMLER) cells, cultured in monolayer systems, was determined using the MTT assay. The IC_50_ values (concentration required to reduce cell viability by 50%) were determined from dose-response curves (Figures S9 and S10) and are presented in Table 1. Both nanoparticle formulations **1:1 NP^15^** and **4:1 NP^15^** displayed nanomolar toxicity towards HMLER and HMLER-shEcad cells. The nanoparticle formulations **1:1 NP^15^** and **4:1 NP^15^** displayed 16- to 23-fold greater potency for CSC-enriched HMLER-shEcad cells than the payload **1**. This shows that encapsulation of **1** into PEG–PLGA (5000:10,000 Da, 1:1 LA:GA) or PEG–PLGA (5000:10,000 Da, 4:1 LA:GA) nanoparticles significantly improves breast CSC cytotoxicity. Interestingly, the **4:1 NP^15^** formulation displays statistically (*p* > 0.05) greater potency towards breast CSCs than bulk breast cancer cells, whereas the **1:1 NP^15^** formulation is equipotent towards breast CSCs and bulk breast cancer cells. Strikingly, the potency of **1:1 NP^15^** and **4:1 NP^15^** towards breast CSCs was 213-fold and 300-fold greater than that of salinomycin, respectively [12]. Salinomycin is an ionophore that was identified as a selective anti-breast CSC agent in a high-throughput screen involving 16,000 bioactive compounds [23]. The empty PEG–PLGA (5000:10,000 Da, 1:1 LA:GA) or PEG–PLGA (5000:10,000 Da, 4:1 LA:GA) nanoparticles were non-toxic towards both HMLER and HMLER-shEcad cells within the concentration range tested (IC_50_ value > 50 µM polymer concentration) (Figures S11 and S12, Table 1). This suggests that the potency of the nanoparticle formations **1:1 NP^15^** and **4:1 NP^15^** is due to their payload **1**, and there is little or no contribution from the PEG–PLGA polymers.

**Figure 4:**
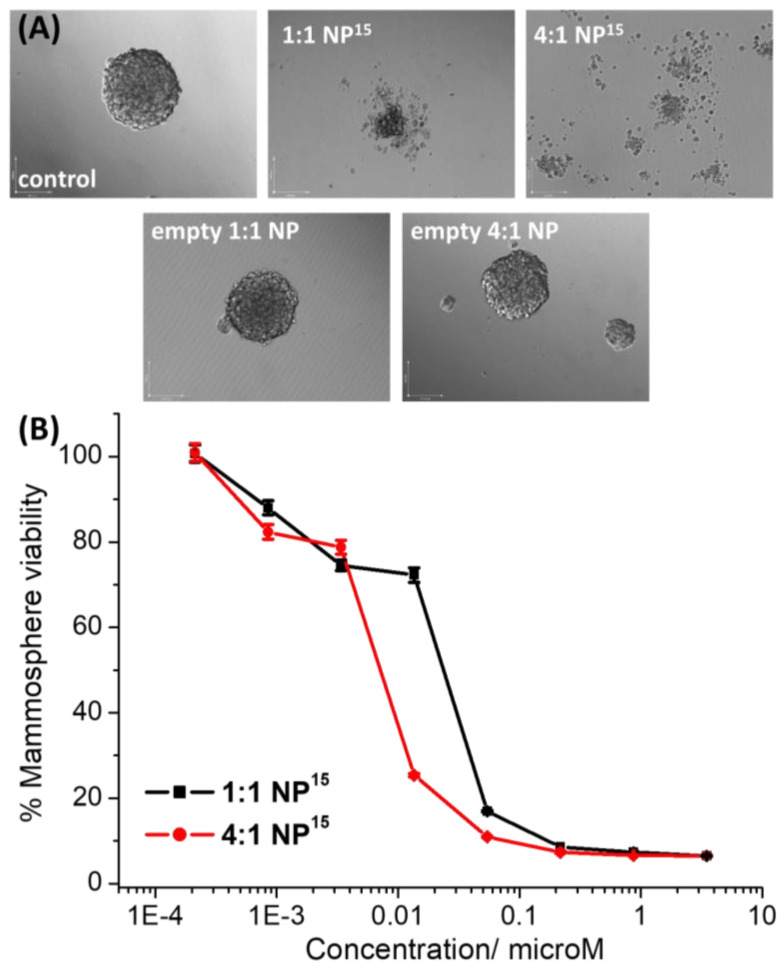
(**A**) Representative bright-field images (×10) of HMLER-shEcad mammospheres in the absence and presence of **1:1 NP^15^** or **4:1 NP^15^** or the empty PEG–PLGA (5000:10,000 Da, 1:1 LA:GA) or PEG–PLGA (5000:10,000 Da, 4:1 LA:GA) nanoparticles at their respective IC_20_ values for 5 days. (**B**) Representative dose-response curves for the treatment of HMLER-shEcad mammospheres with **1:1 NP^15^** or **4:1 NP^15^**.

When breast CSCs are grown in low-attachment conditions with no serum, multicellular structures called mammospheres are formed [24]. Mammospheres are three-dimensional spherical collections of breast CSCs that provide a good model for assessing in vivo potential as they are more representative of the tumour architecture than breast CSCs cultured in monolayers. Mammosphere formation studies, which involve the treatment of single cell suspensions of breast CSCs with a non-lethal dose (IC_20_ value) of a given agent followed by incubation in three-dimensional cell culture conditions for 5 days, revealed that **1:1 NP^15^** and **4:1 NP^15^** both completely disrupted mammosphere formation (Figure 4A). The empty PEG–PLGA (5000:10,000 Da, 1:1 LA:GA) or PEG–PLGA (5000:10,000 Da, 4:1 LA:GA) nanoparticles did not significantly affect the size of mammospheres formed (Figure 4A), indicating that the mammosphere inhibitory effect observed for **1:1 NP^15^** and **4:1 NP^15^** is most likely due the payload **1**. To investigate the effect of **1:1 NP^15^** and **4:1 NP^15^** on mammosphere viability, the TOX8 resazurin-based reagent was employed. The IC_50_ values (concentration required to reduce mammosphere viability by 50%) were interpolated from dose-response curves (Figure 4B) and are summarised in Table 1. The nanoparticle formulations **1:1 NP^15^** and **4:1 NP^15^** exhibited 23-fold and 74-fold greater mammosphere potency than payload **1**, respectively. This shows that encapsulation of **1** into PEG–PLGA (5000:10,000 Da, 1:1 LA:GA) or PEG–PLGA (5000:10,000 Da, 4:1 LA:GA) nanoparticles significantly improves mammosphere cytotoxicity. Interestingly, the **4:1 NP^15^** formulation displays significantly (*p* > 0.05) greater potency towards mammospheres than the **1:1 NP^15^** formulation. Importantly, from a translational perspective, **1:1 NP^15^** and **4:1 NP^15^** were 784-fold and 2534-fold more potent towards mammospheres than salinomycin, respectively [13]. The empty PEG–PLGA (5000:10,000 Da, 1:1 LA:GA) or PEG–PLGA (5000:10,000 Da, 4:1 LA:GA) nanoparticles were non-toxic towards mammospheres within the concentration range tested (IC_50_ value > 33 µM polymer concentration) (Figure S13).

## 3. Conclusions

In summary, we report the encapsulation of a CSC-potent copper(II) complex **1** by compositionally different PEG–PLGA polymers. Specifically, two nanoparticle formulations **1:1 NP^15^** and **4:1 NP^15^** were prepared comprising **1** (15% feed) and PEG–PLGA (5000:10,000 Da, 1:1 LA:GA) or PEG–PLGA (5000:10,000 Da, 4:1 LA:GA), respectively. The nanoparticles formulations **1:1 NP^15^** and **4:1 NP^15^** were shown by DLS and TEM studies to form sphere-like structures with a diameter of around 55–80 nm. Both nanoparticle formulations **1:1 NP^15^** and **4:1 NP^15^** were taken up by breast CSCs to a significant level via an active endocytic mechanism. Notably, the payload release profile and the breast CSC versus bulk breast cancer cell cytotoxicity of **1:1 NP^15^** and **4:1 NP^15^** varied markedly. The nanoparticle formulation with the lower proportion of glycolic acid **4:1 NP^15^** released **1** at a slower, more controlled rate over the course of 72 h in biologically relevant solutions than **1:1 NP^15^**. The nanoparticle formulation **4:1 NP^15^** displayed statistically higher toxicity towards breast CSCs than bulk breast cancer cells (grown in monolayers), whereas **1:1 NP^15^** was equipotent. Importantly, **1:1 NP^15^** and **4:1 NP^15^** were up to 300-fold more toxic towards breast CSCs (grown in monolayers) than salinomycin. The nanoparticle formulation **4:1 NP^15^** exhibited significantly higher potency towards three-dimensionally cultured mammospheres than **1:1 NP^15^** (3-fold), **1** (74-fold), and salinomycin (2534-fold). Our data show that the payload release profile and breast CSC potency of PEG–PLGA nanoparticle formulations (containing **1**) can be modified by changing the ratio of glycolic acid to lactic acid within the PLGA component. These results pave the way for the development of other compositionally diverse PEG–PLGA nanoparticle formulations that can effectively deliver and release anti-CSC metal complexes.

## 4. Materials and Methods

### 4.1. Encapsulation of 1 into PEG–PLGA Nanoparticles

The nanoprecipitation method was used to encapsulate the copper(II) complex **1** into compositionally different PEG–PLGA nanoparticles. Either 10 mg of PEG–PLGA (5000:10,000 Da, 1:1 LA:GA) or PEG–PLGA (5000:10,000 Da, 4:1 LA:GA) and various amounts of **1** (0.5–5 mg), were dissolved in 0.5 mL of DMF. The amount of **1** used varied accordingly to the desired feed, defined as mg of **1**/mg of polymer ×100. The DMF solution was added in a dropwise manner to 5 mL of stirring MilliQ water (0.5 cm magnetic stirrer, 800 rpm rotation speed). The encapsulation reaction was carried out in a 20 mL glass scintillation vial at room temperature. After the addition of the DMF solution (containing the compositionally different PEG–PLGA polymers and **1**) to MilliQ water, the water acquired a milky blue colour due to the Tyndall effect of the nanoparticles formed. At this stage, 4.5 mL of MilliQ water was added to the resultant solution in order to bring the total volume up to 10 mL, and the solution was allowed to stir for an additional 20 min at room temperature. The nanoparticle solution was then loaded onto an Amicon Centrifugal Filtration Device (with a regenerated cellulose membrane and a 100 kDa MW cut-off) and centrifuged for 12 min at 2000 rpm speed (at 18 °C). The concentrated solution was diluted with 10 mL of MilliQ water and centrifuged further under the aforementioned conditions. This was repeated three times to ensure any unencapsulated **1** was removed. The final concentrated suspension was diluted to 1 mL with MilliQ water and filtered to remove any aggregates (a filter with a cut-off of 0.2 μm was used). The filtered suspension was diluted further with MilliQ water and used for further experiments. The amount of copper present in the final suspension was measured by ICP-MS (ThermoScientific ICAP-Qc quadrupole, Waltham, MA, USA). The measured copper content was used to calculate the loading efficiency and encapsulation efficiency; the amount of copper present in the final nanoparticle formulation relative to the amount of polymer (loading efficiency) or **1** (encapsulation efficiency) used ×100. Empty PEG–PLGA (5000:10,000 Da, 1:1 LA:GA) and PEG–PLGA (5000:10,000 Da, 4:1 LA:GA) nanoparticles were prepared using the above method without the addition of **1** and used as controls. In this case, it was assumed that all of the PEG–PLGA (5000:10,000 Da, 1:1 LA:GA) or PEG–PLGA (5000:10,000 Da, 4:1 LA:GA) polymer used (10 mg) formed nanoparticles.

### 4.2. Dynamic Light Scattering and Transmission Electron Microscopy

The nanoparticle size distribution and polydispersity were obtained by loading aqueous solutions of the nanoparticle formulations **1:1 NP^5−50^** or **4:1 NP^5−50^** into disposable micro-cuvettes and measuring the dynamic light scattering (DLS) of the solution using a Zetasizer Nanoseries spectrometer (Malvern Instruments, Malvern, UK). For the transmission electron microscopy (TEM) studies, an aliquot of **1:1 NP^15^** or **4:1 NP^15^** in MilliQ water was allowed to evaporate on a square glass slide and stained with uranyl acetate. Imaging was conducted using a JEOL 2100 Transmission Electron Microscope within the University of Leicester Electron Microscopy Facility (EMF).

### 4.3. Payload Release Studies

The nanoparticle formulations **1:1 NP^15^** or **4:1 NP^15^** were incubated in PBS (pH 7.4) or sodium acetate buffer (pH 5.2) for 72 h at 37 °C. At specific time points over the course of the incubation period, the nanoparticle solution was removed and passed through an Amicon Centrifugal Filter (with a 100 kDa MW cut-off) and replenished with fresh PBS (pH 7.4) or sodium acetate buffer (pH 5.2). The copper content of the filtrates obtained at each of the time points was measured by ICP-MS and used to calculate the percentage of payload released. 

### 4.4. General Cell Culture Conditions

HMLER and HMLER-shEcad cells derived from normal mammary epithelial cells were gifted to us by Prof. R. A. Weinberg (Whitehead Institute, MIT, Cambridge, MA, USA). The cells were cultured using Mammary Epithelial Cell Growth Medium (MEGM) containing BPE, hydrocortisone, hEGF, insulin, and gentamicin/amphotericin-B (Lonza). The cells were handled in a sterile environment at all times and cultured in an incubator that was maintained at 37 °C, with an internal atmosphere containing 5% CO_2_.

### 4.5. Cellular Uptake

Cellular uptake studies involving the nanoparticle formulations **1:1 NP^15^** or **4:1 NP^15^** were conducted under various conditions. HMLER-shEcad cells (*ca.* 1 million) were treated with **1:1 NP^15^** or **4:1 NP^15^** (both 0.15 µM) at 4 °C or 37 °C for 4 h or 8 h. Experiments were also conducted in the presence of endocytosis inhibitor chloroquine (100 μM, 2 h pre-treatment). After incubation, the media containing the nanoparticle formulations **1:1 NP^15^** or **4:1 NP^15^** (with or without chloroquine) were aspirated and the remaining adherent cells were thoroughly washed with 2 mL of PBS, three times. The cells were then collected by trypsinisation and centrifuged to form a pellet. The resultant pellets were digested with 65% HNO_3_ (250 μL) overnight at room temperature. The solutions were then diluted with MilliQ water and measured by ICP-MS to determine the copper content (Thermo Scientific iCAP-Qc quadrupole). The copper content in each sample (cellular material) is represented as Cu (ng) per million cells (overall n = 4).

### 4.6. Cytotoxicity MTT Assay

The colorimetric MTT assay was used to determine the toxicity of the nanoparticle formulations **1:1 NP^15^** and **4:1 NP^15^** and the corresponding empty PEG–PLGA nanoparticles. HMLER and HMLER-shEcad cells (5 × 10^3^) were seeded in each well of a 96-well plate. After incubating the cells overnight, various concentrations of **1:1 NP^15^** or **4:1 NP^15^** or the corresponding empty PEG–PLGA nanoparticles, as determined by ICP-MS, were added and incubated for 72 h (total volume 200 μL). After the incubation period, a PBS solution containing MTT (4 mg/mL) was added to each well of the 96-well plate. Specifically, 20 μL of the PBS-MTT solution was added. After the addition, the 96-well plates were incubated for 4 h. The solution was then removed from each well to leave behind purple formazan crystals. The purple formazan crystals were dissolved in DMSO (200 μL), and the absorbance of the solution was measured using a plate reader at 550 nm. The absorbance of the solution in each well was normalised to untreated control wells and used to generate dose-response curves with a concentration of test agent on the *x*-axis and % HMLER or HMLER-shEcad cell viability on the *y*-axis. The IC_50_ values, the concentration required to reduce cell viability by half, were interpolated from the dose-dependent curves. The cytotoxicity MTT assay was repeated three times per test agent, per cell line. In each experiment, each concentration tested was repeated six times (overall n = 18).

### 4.7. Mammosphere Formation and Viability Assay

HMLER-shEcad cells (5 × 10^3^ cells per well) were plated in ultralow-attachment 96-well plates (Corning) and incubated in MEGM supplemented with B27 (Invitrogen), 20 ng/mL EGF, and 4 μg/mL heparin (Sigma, St. Louis, MO, USA) for 5 days. Studies were also conducted in the presence of **1:1 NP^15^**, **4:1 NP^15^** and the corresponding empty PEG–PLGA nanoparticles. Mammospheres treated with **1:1 NP^15^**, **4:1 NP^15^** or the corresponding empty PEG–PLGA nanoparticles (at their respective IC_20_ values for 5 days) were imaged using a standard inverted microscope within the University of Leicester Advanced Imaging Facility (AIF). The TOX8 solution (20 µL, Sigma) was added to each well to determine the viability of the mammospheres. After the addition, the 96-well plates were incubated for 16 h. The fluorescence of the solution was measured using a plate reader at 590 nm (λ_ex_ = 560 nm). The fluorescence of the solution in each well was normalised to untreated control wells and used to generate dose-response curves with a concentration of test agent on the *x*-axis and % HMLER-shEcad mammosphere viability on the *y*-axis. The IC_50_ values, the concentration required to reduce HMLER-shEcad mammosphere viability by 50%, were interpolated from the dose-response curves. The HMLER-shEcad mammosphere viability assay using TOX8 was repeated three times per test agent. In each experiment, each concentration tested was repeated two times (overall n = 6).

## Figures and Tables

**Table 1:** IC_50_ values of the nanoparticle formulations **1:1 NP^15^** and **4:1 NP^15^**, the empty PEG–PLGA (5000:10,000 Da, 1:1 LA:GA) and PEG–PLGA (5000:10,000 Da, 4:1 LA:GA) nanoparticles, the payload **1**, and salinomycin against HMLER and HMLER-shEcad cells and HMLER-shEcad mammospheres determined after 72 h or 120 h incubation (mean of three independent experiments ± SD).

Compound	HMLER IC_50_ [μM]	HMLER-shEcad IC_50_ [μM]	Mammosphere IC_50_ [μM]
**1:1 NP^15^**	0.0227 ± 0.0006	0.0197 ± 0.0026	0.0236 ± 0.0003
**4:1 NP^15^**	0.0209 ± 0.0021	0.0140 ± 0.0003	0.0073 ± 0.0002
Empty 1:1 NP	>50	>50	>33
Empty 4:1 NP	>50	>50	>33
**1** ^1^	0.21 ± 0.01	0.32 ± 0.02	0.54 ± 0.01
salinomycin ^1^	11.40 ± 0.40	4.20 ± 0.30	18.50 ± 1.50

^1^ Taken from references [12] and [13].

## Data Availability

Not applicable.

## Supplementary Materials

The following supporting information can be downloaded at: https://www.mdpi.com/article/10.3390/molecules28062506/s1, Figure S1. Dynamic light scattering size distribution of 1:1 NP15 suspended in water. Size refers to diameter of nanoparticles in nm.; Figure S2. Dynamic light scattering size distribution of 4:1 NP15 suspended in water. Size refers to diameter of nanoparticles in nm.; Figure S3. Dynamic light scattering size distribution of empty PEG–PLGA (5000:10,000 Da, 1:1 LA:GA) nanoparticles suspended in water. Size refers to diameter of nanoparticles in nm.; Figure S4. Dynamic light scattering size distribution of empty PEG–PLGA (5000:10,000 Da, 4:1 LA:GA) nanoparticles suspended in water. Size refers to diameter of nanoparticles in nm.; Figure S5. Variation in 1:1 NP15 diameter upon incubation in water and mammary epithelial growth medium (MEGM) over the course of 72 h at 37 °C.; Figure S6. Variation in 4:1 NP15 diameter upon incubation in water and mammary epithelial growth medium (MEGM) over the course of 72 h at 37 °C.; Figure S7. Copper content in HMLER-shEcad cells upon pre-incubation with chloroquine (100 µM for 2 h) and then treatment with 1:1 NP15 and 4:1 NP15 (both 0.15 µM for 8 h) at 37 °C.; Figure S8. The amount of copper released from 1:1 NP15 and 4:1 NP15 upon incubation in sodium acetate buffer (pH 5.2) over the course of 72 h at 37 °C.; Figure S9. Representative dose-response curves for the treatment of HMLER and HMLER-shEcad cells with 1:1 NP15.; Figure S10. Representative dose-response curves for the treatment of HMLER and HMLER-shEcad cells with 4:1 NP15.; Figure S11. Representative dose-response curves for the treatment of HMLER and HMLER-shEcad cells with empty PEG–PLGA (5000:10,000 Da, 1:1 LA:GA) nanoparticles.; Figure S12. Representative dose-response curves for the treatment of HMLER and HMLER-shEcad cells with empty PEG–PLGA (5000:10,000 Da, 4:1 LA:GA) nanoparticles.; Figure S13. Representative dose-response curves for the treatment of HMLER-shEcad mammospheres with empty PEG–PLGA (5000:10,000 Da, 1:1 LA:GA) nanoparticles or empty PEG–PLGA (5000:10,000 Da, 4:1 LA:GA) nanoparticles.

## Sample Availability

Samples of the compounds are available from the authors.
