# Supplementary material for: Payload Release Profile and Anti-Cancer Stem Cell Properties of Compositionally Different Polymeric Nanoparticles Containing a Copper(II) Complex
Source: Molecules. 2023 Mar 9;28(6):2506. doi: 10.3390/molecules28062506 (PMC10051418; doi:10.3390/molecules28062506)
Supplement: Supplementary file 1 [file molecules-28-02506-s001.zip › molecules-2227824-supplementary.pdf]

Supporting Information for

Payload Release Profile and Anti-Cancer Stem Cell Properties of Compositionally Different Polymeric Nanoparticles Containing a Copper(II) Complex

Ginevra Passeri, Joshua Northcote-Smith and Kogularamanan Suntharalingam *

School of Chemistry, University of Leicester, Leicester LE1 7RH, UK

* Correspondence: k.suntharalingam@leicester.ac.uk; Tel.: +44-(0)116-294-4562

Table of Content

Figure S1. Dynamic light scattering size distribution of **1:1 NP[15]** suspended in water. Size refers to diameter of nanoparticles in nm.

Figure S2. Dynamic light scattering size distribution of **4:1 NP[15]** suspended in water. Size refers to diameter of nanoparticles in nm.

Figure S3. Dynamic light scattering size distribution of empty PEG–PLGA (5000:10,000 Da, 1:1 LA:GA) nanoparticles suspended in water. Size refers to diameter of nanoparticles in nm.

Figure S4. Dynamic light scattering size distribution of empty PEG–PLGA (5000:10,000 Da, 4:1 LA:GA) nanoparticles suspended in water. Size refers to diameter of nanoparticles in nm.

Figure S5. Variation in **1:1 NP[15]** diameter upon incubation in water and mammary epithelial growth medium (MEGM) over the course of 72 h at 37 °C.

Figure S6. Variation in **4:1 NP[15]** diameter upon incubation in water and mammary epithelial growth medium (MEGM) over the course of 72 h at 37 °C.

Figure S7. Copper content in HMLER-shEcad cells upon pre-incubation with chloroquine (100 μM for 2 h) and then treatment with **1:1 NP[15]** and **4:1 NP[15]** (both 0.15 μM for 8 h) at 37 °C.

Figure S8. The amount of copper released from **1:1 NP[15]** and **4:1 NP[15]** upon incubation in sodium acetate buffer (pH 5.2) over the course of 72 h at 37 °C.

Figure S9. Representative dose-response curves for the treatment of HMLER and HMLER-shEcad cells with **1:1 NP[15]**.

Figure S10. Representative dose-response curves for the treatment of HMLER and HMLER-shEcad cells with **4:1 NP[15]**.

Figure S11. Representative dose-response curves for the treatment of HMLER and HMLER-shEcad cells with empty PEG–PLGA (5000:10,000 Da, 1:1 LA:GA) nanoparticles.

Figure S12. Representative dose-response curves for the treatment of HMLER and HMLER-shEcad cells with empty PEG–PLGA (5000:10,000 Da, 4:1 LA:GA) nanoparticles.

Figure S13. Representative dose-response curves for the treatment of HMLER-shEcad mammospheres with empty PEG–PLGA (5000:10,000 Da, 1:1 LA:GA) nanoparticles or empty PEG–PLGA (5000:10,000 Da, 4:1 LA:GA) nanoparticles.

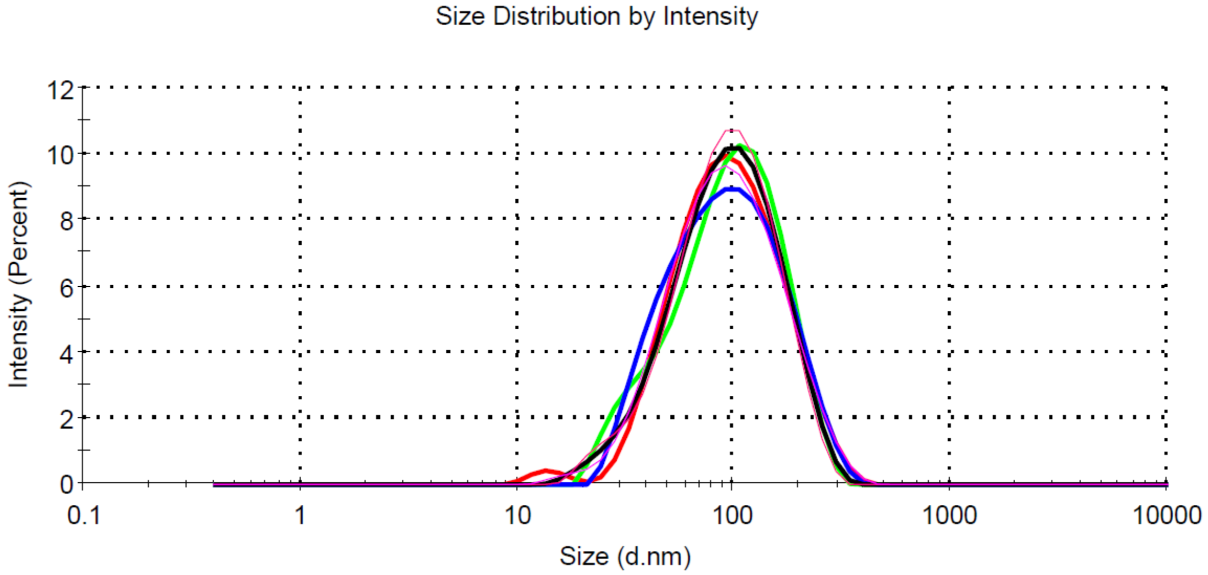

Figure S1. Dynamic light scattering size distribution of **1:1 NP**[15] suspended in water. Size refers to diameter of nanoparticles in nm.

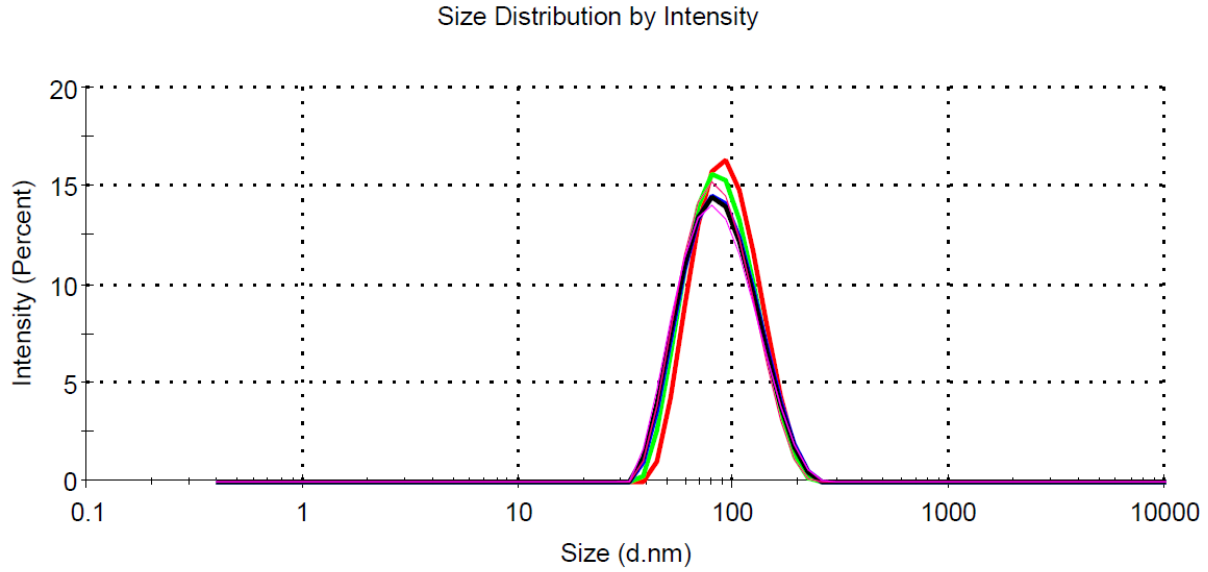

Figure S2. Dynamic light scattering size distribution of **4:1 NP**[15] suspended in water. Size refers to diameter of nanoparticles in nm.

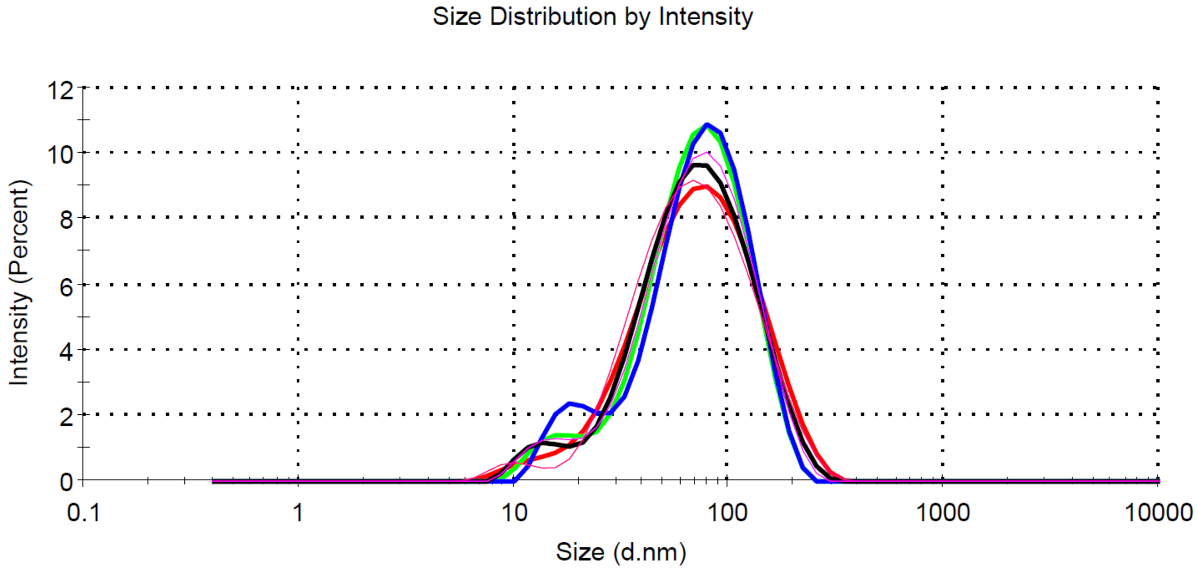

Figure S3. Dynamic light scattering size distribution of empty PEG–PLGA (5000:10,000 Da, 1:1 LA:GA) nanoparticles suspended in water. Size refers to diameter of nanoparticles in nm.

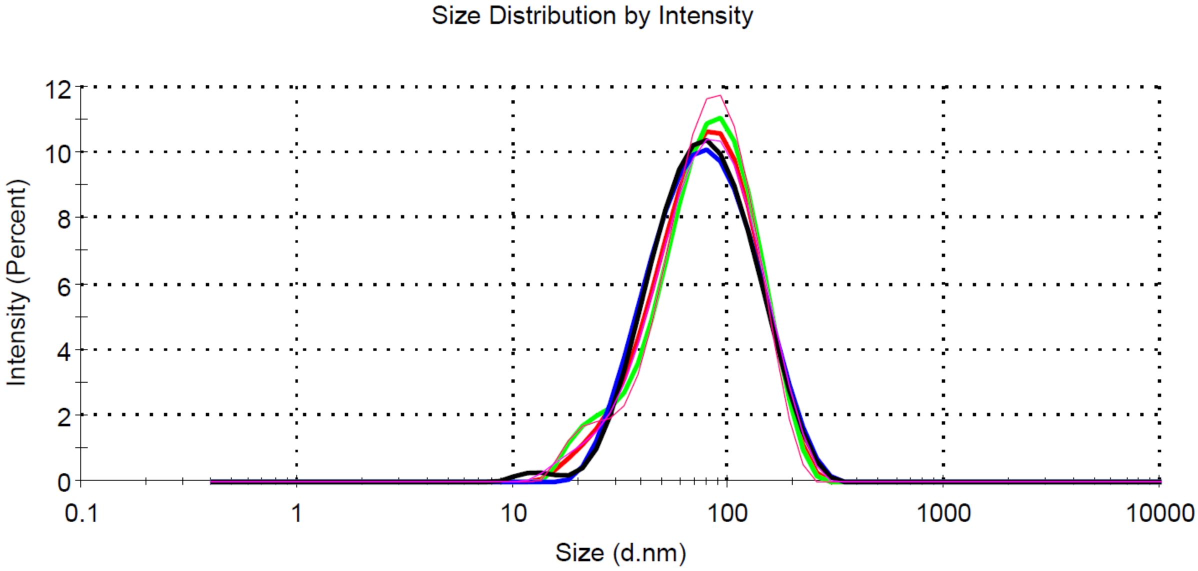

Figure S4. Dynamic light scattering size distribution of empty PEG–PLGA (5000:10,000 Da, 4:1 LA:GA) nanoparticles suspended in water. Size refers to diameter of nanoparticles in nm.

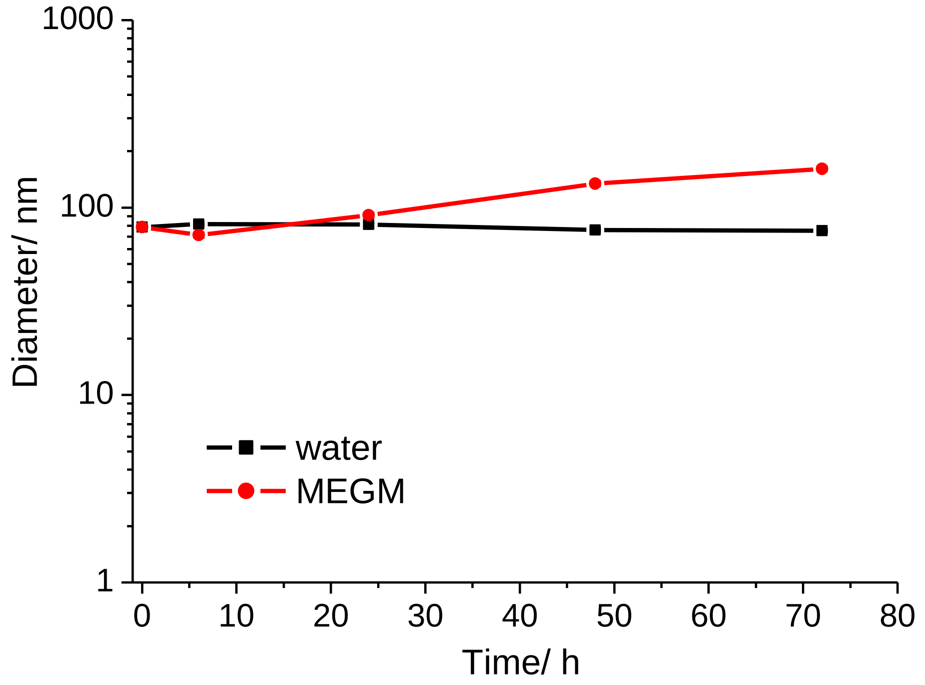

Figure S5. Variation in **1:1 NP[15]** diameter upon incubation in water and mammary epithelial growth medium (MEGM) over the course of 72 h at 37 °C.

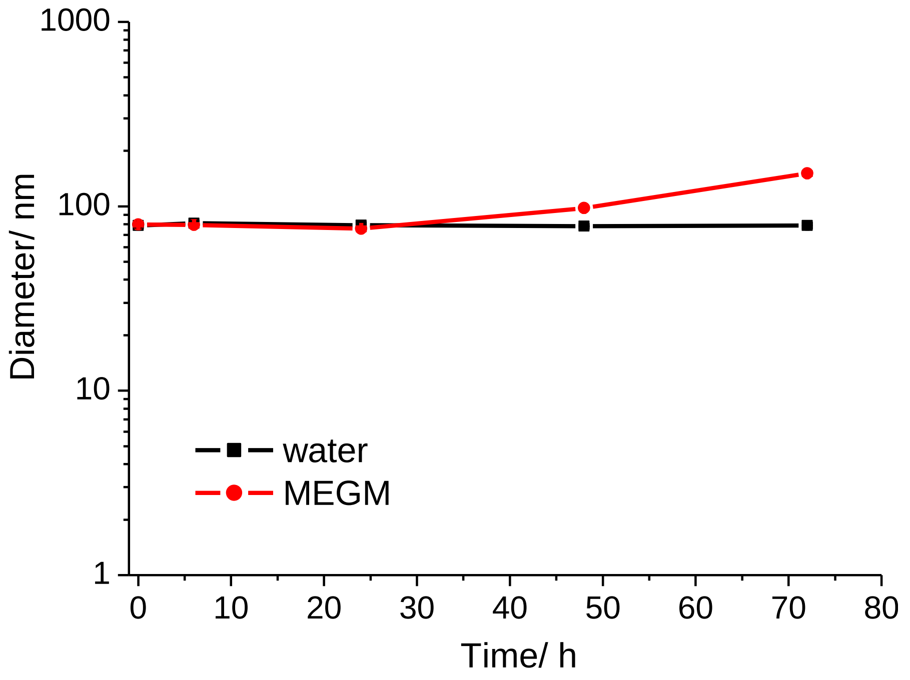

Figure S6. Variation in **4:1 NP[15]** diameter upon incubation in water and mammary epithelial growth medium (MEGM) over the course of 72 h at 37 °C.

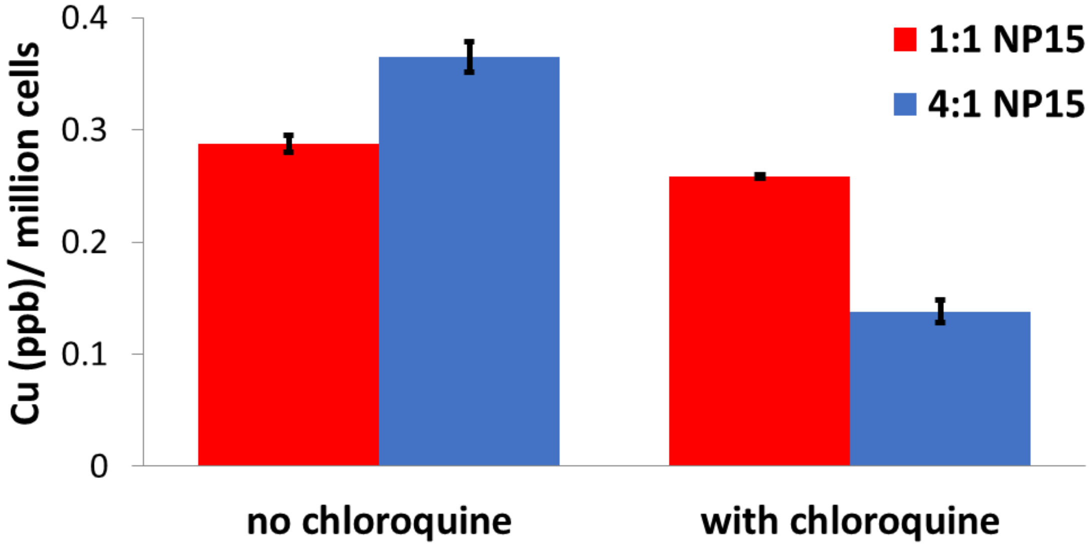

Figure S7. Copper content in HMLER-shEcad cells upon pre-incubation with chloroquine (100 µM for 2 h) and then treatment with **1:1 NP**^{15} and **4:1 NP**^{15} (both 0.15 µM for 8 h) at 37 °C.

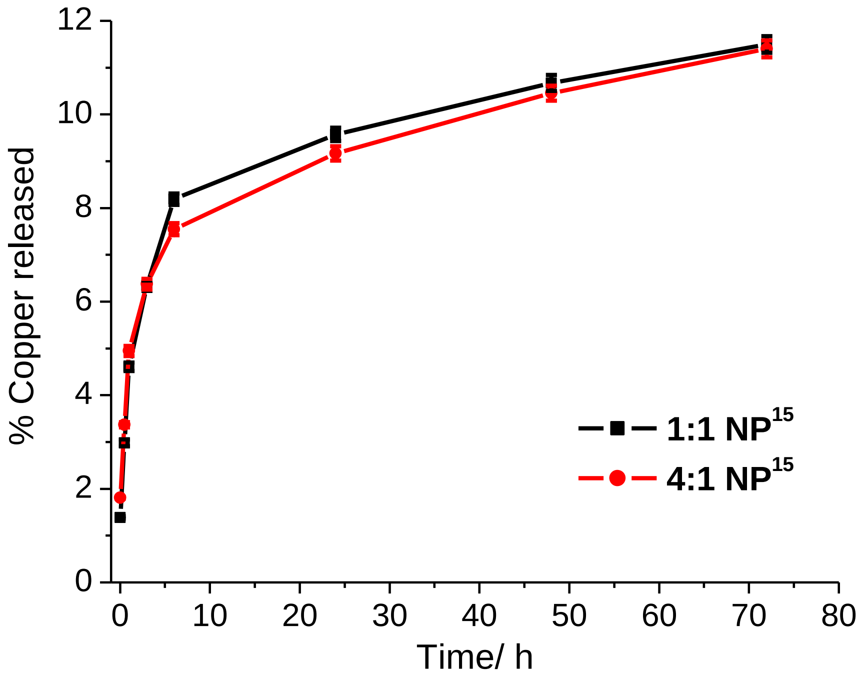

Figure S8. The amount of copper released from **1:1 NP**^{15} and **4:1 NP**^{15} upon incubation in sodium acetate buffer (pH 5.2) over the course of 72 h at 37 °C.

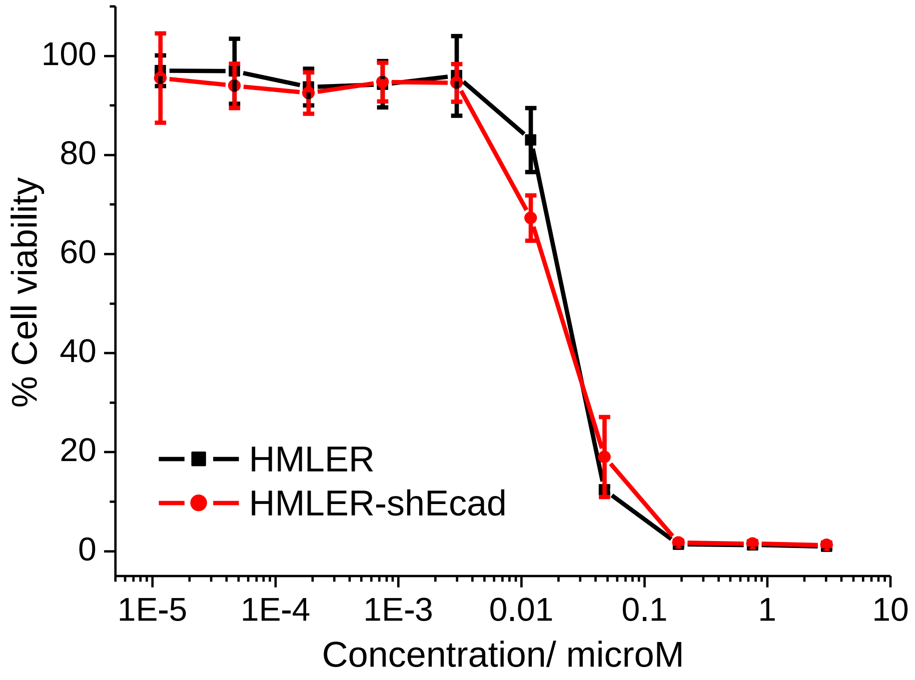

Figure S9. Representative dose-response curves for the treatment of HMLER and HMLER-shEcad cells with **1:1 NP[15]**.

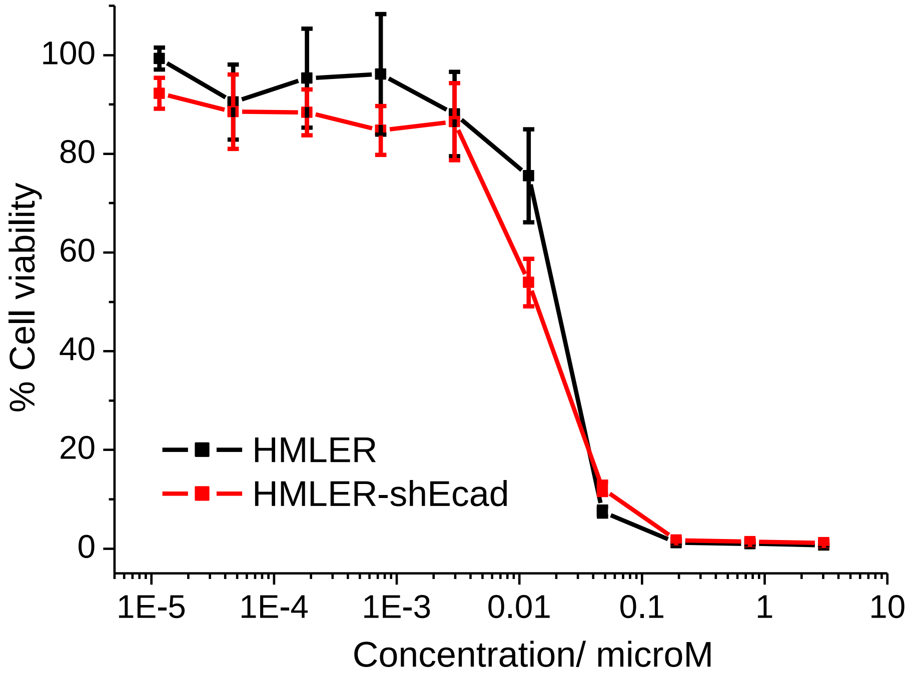

Figure S10. Representative dose-response curves for the treatment of HMLER and HMLER-shEcad cells with **4:1 NP[15]**.

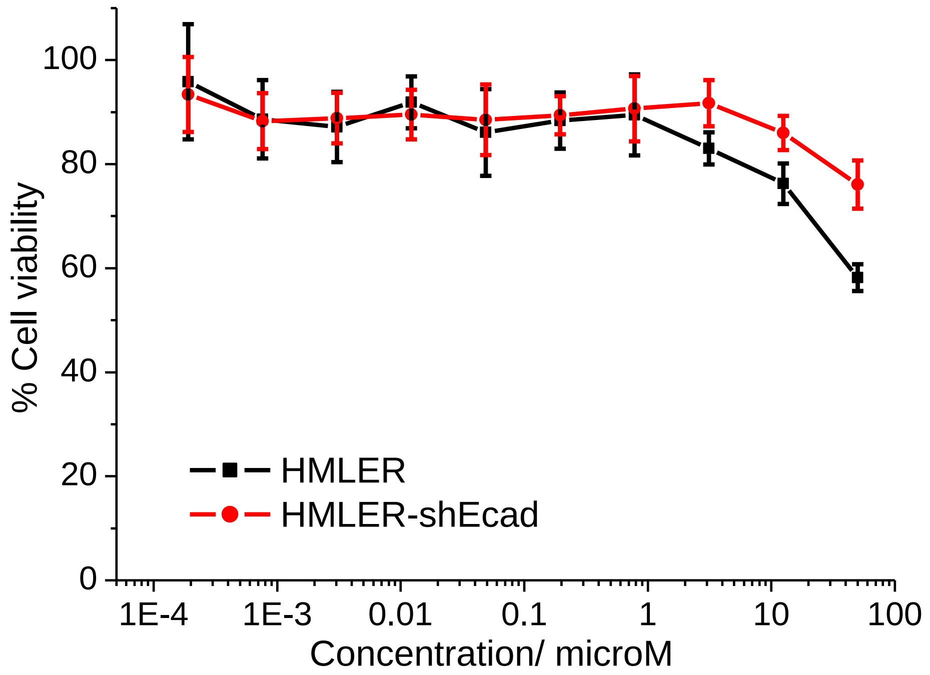

Figure S11. Representative dose-response curves for the treatment of HMLER and HMLER-shEcad cells with empty PEG–PLGA (5000:10,000 Da, 1:1 LA:GA) nanoparticles.

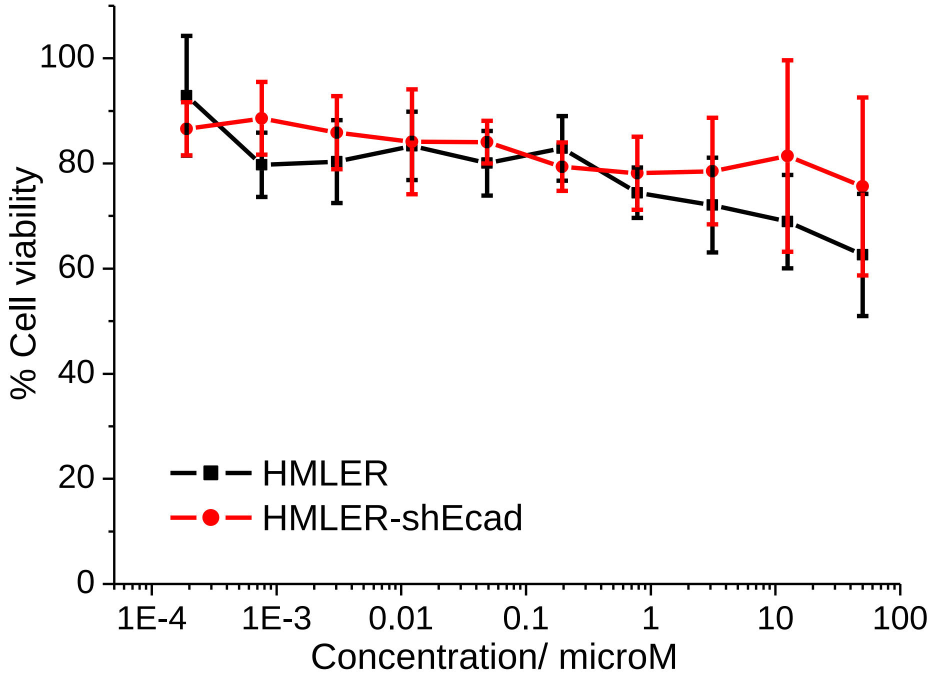

Figure S12. Representative dose-response curves for the treatment of HMLER and HMLER-shEcad cells with empty PEG–PLGA (5000:10,000 Da, 4:1 LA:GA) nanoparticles.

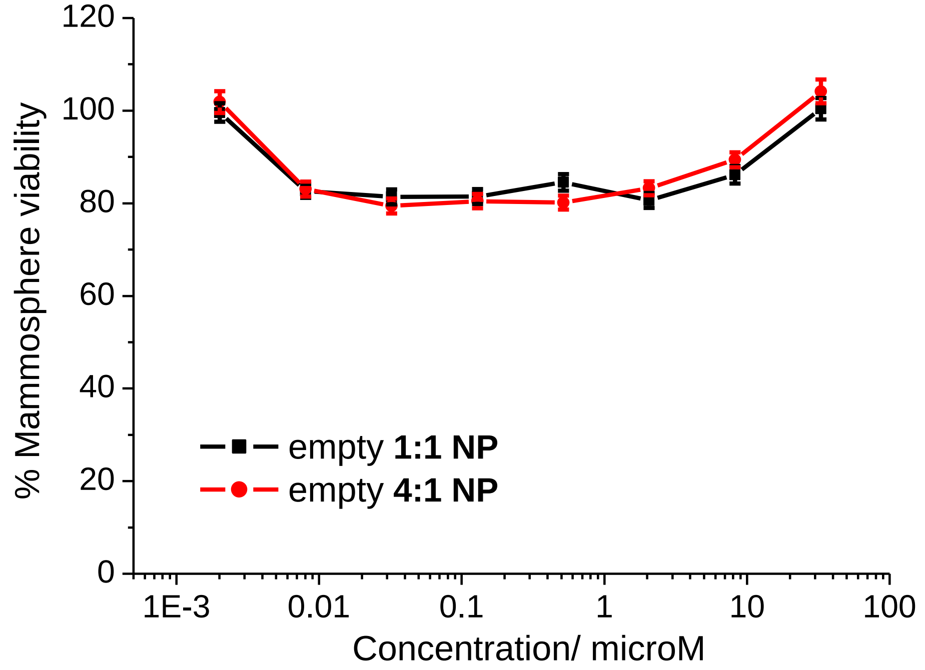

Figure S13. Representative dose-response curves for the treatment of HMLER-shEcad mammospheres with empty PEG–PLGA (5000:10,000 Da, 1:1 LA:GA) nanoparticles or empty PEG–PLGA (5000:10,000 Da, 4:1 LA:GA) nanoparticles.
